# Management of Implant Failure in Long Bones and Its Functional Outcomes

**DOI:** 10.7759/cureus.105877

**Published:** 2026-03-26

**Authors:** Harjit K Chawla, Jagdeep S Rehncy, Jaskaran Bhatti, Mukul Sharma, Amandeep S Bakshi

**Affiliations:** 1 Orthopaedics, Government Medical College Patiala, Patiala, IND

**Keywords:** broken implant retrieval, implant failure, nailing, non union, plating

## Abstract

Background: Implant failure in long bone fractures remains a significant complication in orthopaedic trauma practice and reflects an imbalance between mechanical stability and biological healing.

Objective: To evaluate etiological factors, radiological findings, management strategies, and functional outcomes following revision surgery for implant failure.

Methods: This prospective observational study included 50 adult patients with implant failure. Nonunion severity was assessed using the Nonunion Scoring System (NUSS). Pain was assessed using the Visual Analog Scale (VAS), and functional outcome was evaluated using the Katz Activities of Daily Living (ADL) score. Statistical analysis was performed using the Statistical Package for the Social Sciences (SPSS), version 20.0 (IBM Corp., Armonk, NY). Pearson correlation coefficient was applied for association analysis, with a p-value < 0.05 considered statistically significant.

Results: The majority of patients were aged 31-50 years with male predominance (56%). Open fractures constituted 60% of cases. The tibia was the most commonly involved bone (32%). Implant breakage (26%) and loosening (22%) were the predominant modes of failure. Most failures occurred between three and six months (40%). The mean VAS score improved from 3.6 ± 1.2 preoperatively to 0.3 ± 0.2 at six months (p < 0.001). Eighty-two percent of patients achieved good-to-excellent functional outcomes.

Conclusion: Implant failure is multifactorial in origin. A structured revision approach addressing both mechanical and biological factors results in favorable functional recovery.

## Introduction

Long bone fractures are common injuries worldwide and contribute significantly to trauma-related morbidity [1]. Although modern fixation techniques have improved fracture stabilization, implant failure remains a challenging complication [2]. Implant failure encompasses breakage, loosening, bending, or loss of fixation prior to fracture union [3]. According to strain theory and the diamond concept, successful fracture healing requires both mechanical stability and biological integrity [4,5]. Mechanical contributors include inadequate fixation, improper working length, and premature loading [6]. Biological factors such as open fractures, infection, smoking, diabetes mellitus, and osteoporosis impair fracture healing [7,8]. Open fractures and tibial injuries are particularly susceptible due to compromised vascularity [9]. Revision surgery must therefore address both mechanical stability and biological enhancement to optimize outcomes [10]. This study evaluates implant failure patterns and functional outcomes in a tertiary care population.

## Materials and methods

This prospective observational study was conducted at a tertiary care center between January 2024 and December 2024 after obtaining approval from the Institutional Ethics Committee at Government Medical College Patiala (Approval No: Trg8(109)2024/28172-189). A consecutive sampling technique was employed, and all eligible patients presenting during the study period were enrolled. Inclusion criteria were: adult patients aged ≥18 years, patients presenting with implant failure following internal fixation of long bone fractures (humerus, femur, tibia, radius, or ulna), radiological evidence of implant failure (breakage, loosening, bending, or loss of fixation), patients willing to undergo revision surgery, and patients providing written informed consent. Exclusion criteria were: pathological fractures (e.g., malignancy-related fractures), periprosthetic fractures, patients with active systemic infection unrelated to the fracture site, patients medically unfit for surgery, and patients lost to follow-up before completion of six months.

Open fractures were classified according to the Gustilo-Anderson classification system [11]. Nonunion severity was assessed using the Nonunion Scoring System (NUSS) [12]. Pain was evaluated using the Visual Analog Scale (VAS), a validated tool for measuring pain intensity consisting of a 10-cm horizontal line anchored by “no pain” and “worst imaginable pain” [13]. Functional independence was assessed using the Katz Index of Independence in Activities of Daily Living (Katz ADL) [14]. Permission to use the Katz ADL was obtained for not-for-profit educational and research purposes, and the Hartford Institute for Geriatric Nursing, New York University, College of Nursing, is acknowledged as the source. Patients underwent revision surgery based on mechanical and biological assessment and were followed for a minimum of six months postoperatively.

Outcome measures

Primary Outcome

The primary outcome measure was functional outcome at six months following revision surgery, assessed using the Katz Index of Independence in Activities of Daily Living (Katz ADL) score. Outcome grading was defined as: 1. Score 6 = Excellent, 2. Score 5 = Good, 3. Score 3-4 = Fair, 4. Score 0-2 = Poor.

Secondary Outcome

Secondary outcome measures included: 1. Pain reduction following revision surgery, assessed using the VAS, 2. Radiological evidence of fracture union at six months, 3. Pattern and timing of implant failure.

Timing of Assessments

VAS scores were recorded: 1. Preoperatively (at the presentation with implant failure), 2. At six weeks postoperatively, 3. At three months, 4. At six months postoperatively. Katz ADL scores were assessed: 1. Preoperatively, 2. At three months, 3. At six months postoperatively (final outcome assessment). Radiological evaluation for fracture union was performed at three and six months using standard anteroposterior (AP) and lateral radiographs. Radiological union was defined as the presence of bridging callus across at least three out of four cortices on anteroposterior and lateral radiographs

Statistical analysis

Statistical analysis was performed using the Statistical Package for the Social Sciences (SPSS), version 20.0 (IBM Corp., Armonk, NY, USA). Continuous variables such as age, duration to implant failure, preoperative and postoperative VAS scores, and Nonunion Scoring System (NUSS) scores were expressed as mean ± standard deviation (SD). Categorical variables, including sex, fracture type, bone involved, implant failure pattern, management strategy, postoperative complications, and functional outcome categories, were expressed as frequency and percentage. Pre-operative and post-operative pain scores (VAS) were compared using a paired t-test. A p-value less than 0.05 was considered statistically significant. Descriptive statistics were used to summarize changes in pain and functional outcomes over the follow-up period.

## Results

Demographic characteristics

Table 1 shows that implant failure was most common in the 31-40-year age group (30%), followed by the 41-50-year age group (24%).

**Table 1:** Age distribution Total verified = 50

Category	Value (n=50)	%
≤30	9	18%
31–40	15	30%
41–50	12	24%
51–60	6	12%
>60	8	16%
Total	50	100%

The study included 50 patients. Implant failure was most common in the 31-40-year age group (30%), followed by the 41-50-year age group (24%) (Table 1).

Injury characteristics and primary fixation details

 Table 2 shows that open fractures accounted for 60% of cases.

**Table 2:** Nature of fracture Open fractures total = 30 (60%).

Category	Value (n=50)	%
Closed	20	40%
Open I	13	26%
Open II	11	22%
Open III	6	12%
Total	50	100%

 Table 3 shows that the tibia (32%) was the most frequently involved bone, followed by the femur (28%).

**Table 3:** Bone involved

Category	Value (n=50)	%
Tibia	16	32%
Femur	14	28%
Humerus	11	22%
Radius/Ulna	9	18%
Total	50	100%

Table 4 shows that plates (38%) and intramedullary nails (32%) were the most commonly used implants in primary surgery.

**Table 4:** Initial implant used

Category	Value (n=50)	%
Plate	19	38%
IM Nail	16	32%
External Fixator	11	22%
K-wire	4	8%
Total	50	100%

Table 5 shows that most implant failures occurred between three and six months (40%). 

**Table 5:** Time to failure

Category	Value (n=50)	%
<3 months	15	30%
3–6 months	20	40%
6–12 months	12	24%
>12 months	3	6%
Total	50	100%

Open fractures accounted for 60% of cases (Table 2). The tibia was the most frequently involved bone (32%) (Table 3). Plates (38%) and intramedullary nails (32%) were the most commonly used implants (Table 4). Most implant failures occurred between three and six months (40%) (Table 5).

Implant failure pattern and radiological findings

Table 6 shows that the implant breakage (26%) was the most common failure pattern, followed by loosening (22%).

**Table 6:** Implant failure type

Category	Value (n=50)	%
Breakage	13	26%
Loosening	11	22%
Plate Bending	10	20%
Nail Breakage	8	16%
Backing Out	8	16%
Total	50	100%

Table 7 shows that radiological abnormalities were documented in 20 out of 50 patients (40%). Among these, migration (35%) and malalignment (30%) were the most frequent findings. Multiple radiological findings were observed in some patients.

**Table 7:** Distribution of radiological abnormalities among patients with implant failure (n = 20) Percentages are calculated based on patients with radiological abnormalities (n = 20).

Category	Value (n=20)	%
Migration	7	35%
Malalignment	6	30%
Bone Loss	4	20%
Nonunion	3	15%
Total Reported Findings	20	

Nonunion severity and management strategy

Table 8 shows that most patients were classified as mild (42%) or moderate (34%) severity. 

**Table 8:** Non union severity score (NUSS) category

Category	Value (n=50)	%
Mild	21	42%
Moderate	17	34%
Severe	11	22%
Very Severe	1	2%
Total	50	100%

Table 9 shows that Ilizarov fixation (26%) and revision plating (24%) were the most commonly performed revision procedures.

**Table 9:** Management strategy

Category	Value (n=50)	%
Ilizarov	13	26%
Revision Plate	12	24%
Exchange Nail	9	18%
Bone Graft	9	18%
Combined	7	14%
Total	50	100%

Most patients were classified as mild (42%) or moderate (34%) severity according to the nonunion scoring system (Table 8). Ilizarov fixation (26%) and revision plating (24%) were the most commonly performed revision procedures (Table 9).

Postoperative complications 

Table 10 shows that infection was observed in 6% of cases. Ninety percent of patients had no postoperative complications.

**Table 10:** Post operative complications

Category	Value (n=50)	%
Infection	3	6%
Delayed Union	1	2%
Limb Shortening	1	2%
None	45	90%
Total	50	100%

The mean follow-up duration was 6.4 ± 0.8 months (range: 6-8 months). Postoperative complications were assessed during the six-month follow-up period. Infection was observed in three patients (6%), delayed union in one patient (2%), and limb shortening in one patient (2%). Forty-five patients (90%) had no postoperative complications at six months. The mean VAS score decreased from 3.6 ± 1.2 preoperatively to 0.3 ± 0.2 at six months postoperatively. This improvement was statistically significant using a paired t-test (p < 0.001).

Functional outcomes

Table 11 shows that most patients achieved good (46%) or excellent (36%) functional outcomes.

**Table 11:** Functional outcomes

Category	Value (n=50)	%
Excellent	18	36%
Good	23	46%
Fair	8	16%
Poor	1	2%
Total	50	100%

Functional outcome was evaluated at six months postoperatively using the Katz Activities of Daily Living (ADL) score. At final follow-up, 18 patients (36%) achieved an excellent outcome (score 6) and 23 patients (46%) achieved a good outcome (score 5). Eight patients (16%) had fair outcomes, and 1 patient (2%) had a poor outcome.

## Discussion

The present study evaluated implant failure patterns and functional outcomes following revision surgery in adult patients with long bone fractures. Implant failure was most frequently observed in patients aged 31-50 years. This age distribution is consistent with epidemiological findings reported by Zura et al., who described higher rates of fracture-related complications among younger, economically active individuals exposed to high-energy trauma [7]. Open fractures accounted for 60% of cases in the present study. Previous literature has reported higher complication rates in open fractures, likely due to soft-tissue compromise and contamination [9]. Similarly, Metsemakers et al. emphasized the role of biological impairment and fracture-related infection in nonunion [8]. In our cohort, the high proportion of open injuries was associated with implant failure; however, a causal relationship cannot be established given the observational study design.

The tibia was the most frequently involved bone (32%) in our series. Prior studies have reported higher nonunion rates in tibial fractures compared to other long bones [7]. The anatomical characteristics of the tibia, including relatively limited soft tissue coverage, may be associated with delayed healing; however, our findings demonstrate distribution rather than a causal association. Implant breakage (26%) and loosening (22%) were the predominant failure patterns observed. Biomechanical principles described by Perren SM [3] and Claes et al. [6] indicate that sustained cyclic loading in the setting of inadequate biological healing may contribute to hardware fatigue. While our findings are consistent with these principles, the present study design does not allow for the determination of direct causality.

Most implant failures occurred between three and six months following primary fixation. This period corresponds to the expected consolidation phase of fracture healing, as described in the diamond concept by Giannoudis et al. [2]. The temporal distribution observed in our study suggests an association between the consolidation phase and the occurrence of implant failure. The use of the Nonunion Scoring System (NUSS) [12] facilitated structured stratification of cases based on mechanical and biological parameters. Patients categorized as mild or moderate severity demonstrated favorable functional outcomes following revision procedures. These findings indicate that systematic assessment may be associated with improved clinical planning, although definitive causal inference cannot be drawn.

Functional outcomes were favorable, with 82% of patients achieving good-to-excellent results at six months. Similar outcomes have been reported in studies emphasizing restoration of alignment and biological optimization in revision surgery [4]. Pain scores improved significantly following revision procedures, with mean VAS decreasing from 3.6 ± 1.2 to 0.3 ± 0.2 (p < 0.001). This improvement is associated with mechanical stabilization and biological augmentation; however, causation cannot be conclusively established. Postoperative complication rates were low, with infection observed in 6% of cases. Published literature reports infection rates between 5-10% in similar clinical contexts [8,9]. Our findings are comparable to these reports.

Study limitations

The present study has several limitations. First, the sample size was relatively small (n = 50), which may limit statistical power and reduce the ability to detect less common patterns or subgroup differences. Second, the study was conducted at a single tertiary care center, which may limit the generalizability of the findings to other healthcare settings with different patient demographics, surgical expertise, or resource availability. Third, as a prospective observational study employing consecutive sampling, the potential for selection bias cannot be completely excluded. Patients presenting to a tertiary referral center may represent more complex or advanced cases of implant failure, which could influence the observed distribution of fracture types and failure patterns.

Additionally, the follow-up duration was limited to six months. Although this period was sufficient to assess early functional recovery and radiological union, longer follow-up would be necessary to evaluate long-term functional outcomes, late complications, and durability of revision procedures. Finally, due to the observational design, causal relationships between risk factors and implant failure cannot be definitively established. The findings should therefore be interpreted as associative rather than causal.

## Conclusions

Implant failure in long bones represents a complex clinical problem associated with both mechanical instability and compromised biological healing. In the present prospective observational study, implant failure was most commonly observed in tibial fractures and open injuries, with implant breakage and loosening constituting the predominant failure patterns. The majority of failures occurred within three to six months following primary fixation, corresponding to the fracture consolidation phase.

Structured preoperative assessment using the NUSS facilitated objective stratification of mechanical and biological factors, allowing individualized revision planning. Revision procedures addressing both stability and biological optimization were associated with significant pain reduction and favorable functional recovery, with 82% of patients achieving good-to-excellent outcomes at six months.

Although the findings suggest that systematic evaluation and tailored revision strategies are associated with improved short-term outcomes, causal inferences cannot be drawn due to the observational design. These results underscore the importance of comprehensive mechanical and biological assessment in the management of implant failure. Further multicentric studies with larger sample sizes and longer follow-up durations are warranted to validate these findings, refine risk-stratification models, and establish standardized management protocols for implant failure in long-bone fractures.
